# A New Method for Species Identification via Protein-Coding and Non-Coding DNA Barcodes by Combining Machine Learning with Bioinformatic Methods

**DOI:** 10.1371/journal.pone.0030986

**Published:** 2012-02-20

**Authors:** Ai-bing Zhang, Jie Feng, Robert D. Ward, Ping Wan, Qiang Gao, Jun Wu, Wei-zhong Zhao

**Affiliations:** 1 College of Life Sciences, Capital Normal University, Beijing, People's Republic of China; 2 School of Mathematical Sciences, Capital Normal University, Beijing, People's Republic of China; 3 Wealth from Oceans Flagship, CSIRO Marine and Atmospheric Research, Hobart, Tasmania, Australia; Brigham and Women's Hospital, United States of America

## Abstract

Species identification via DNA barcodes is contributing greatly to current bioinventory efforts. The initial, and widely accepted, proposal was to use the protein-coding cytochrome c oxidase subunit I (COI) region as the standard barcode for animals, but recently non-coding internal transcribed spacer (ITS) genes have been proposed as candidate barcodes for both animals and plants. However, achieving a robust alignment for non-coding regions can be problematic. Here we propose two new methods (DV-RBF and FJ-RBF) to address this issue for species assignment by both coding and non-coding sequences that take advantage of the power of machine learning and bioinformatics. We demonstrate the value of the new methods with four empirical datasets, two representing typical protein-coding COI barcode datasets (neotropical bats and marine fish) and two representing non-coding ITS barcodes (rust fungi and brown algae). Using two random sub-sampling approaches, we demonstrate that the new methods significantly outperformed existing Neighbor-joining (NJ) and Maximum likelihood (ML) methods for both coding and non-coding barcodes when there was complete species coverage in the reference dataset. The new methods also out-performed NJ and ML methods for non-coding sequences in circumstances of potentially incomplete species coverage, although then the NJ and ML methods performed slightly better than the new methods for protein-coding barcodes. A 100% success rate of species identification was achieved with the two new methods for 4,122 bat queries and 5,134 fish queries using COI barcodes, with 95% confidence intervals (CI) of 99.75–100%. The new methods also obtained a 96.29% success rate (95%CI: 91.62–98.40%) for 484 rust fungi queries and a 98.50% success rate (95%CI: 96.60–99.37%) for 1094 brown algae queries, both using ITS barcodes.

## Introduction

DNA barcoding has become increasingly popular as a tool for species discrimination and identification [1]–[19], although some aspects remain controversial [20]–[34]. As of October 2011, there were 1, 381, 970 barcodes from 114, 873 species in the Barcode of Life Database (BOLD, www.barcodinglife.org), covering a very wide spectrum of species from algae, fungi, bacteria and plants to invertebrates and vertebrates. The COI barcode has proven to be a successful species-discriminator in most animal groups, but is generally less successful elsewhere. BOLD therefore also includes internal transcribed spacer (ITS) sequences for fungal identification and the two chloroplast-encoded genes ribulose bisphosphate carboxylase (rbcL) and maturaseK (MatK) for plants.

A fundamental issue in DNA barcoding is how best to assign a query sequence from an unknown specimen to the correct species in the reference sequence database [15], [19], [24], [25], [35]–[43]. Currently, most empirical studies employ traditional phylogenetic methods such as Neighbour-joining [1], [2], [44] to construct an evolutionary tree with both query and reference sequences. A sequence visually falling in a single-species clade is treated as the conspecific of that species. However, if the query falls into a polyphyletic or paraphyletic clade, assignation to correct species becomes ambiguous.

More recently, other statistical approaches to assignation have been suggested including decision theory [11] and Bayesian methods [36], [38], [39]. Zhang and colleagues have proposed a neural network based approach [15], [45]. Neural networks were originally developed to model the function of connected neurons in the brain [46]. However, their utility as a general computational tool was realized with the development of the back-propagation method [47]–[50]. It has been applied successfully in many fields, including speech synthesis, handwriting recognition and medical diagnostics. In molecular genetics it has been applied to some aspects of DNA/RNA and protein sequence analysis [51], [52], such as protein and ribosomal RNA classification [53]–[55] and phylogenetic reconstruction [56]. Some machine learning techniques have also been proposed for the analysis of DNA sequences, including Classification and Regression Trees (CART) [57], [58], Random Forest (RF) [58], [59], and Support Vector Machines [60]. All these methods, and those based on tree construction, require a prior alignment of sequences. Sequence alignment is generally straight-forward for protein-coding regions, such as the COI sequence proposed as the universal animal barcode, but can be difficult when barcodes are based on non-coding regions such as 28 S or ITS which have variable length and indels (gaps). A robust alignment of non-coding regions can be extremely hard to achieve. Even if an alignment can be obtained using existing algorithms, such as those employed in ClustalW (http://www.clustal.org/) [61], the computation of genetic distances among sequences is still problematic since there is, so far, no molecular evolutionary model which simulates the evolution of DNA sequences with indels. The indels are generally removed or treated as missing data in the subsequent analysis. Sometimes, indels may be coded as fifth states or given other codes, introducing extra assumptions. While it is necessary, for some taxa, to incorporate non-coding barcodes into the BOLD system, it would be advantageous to eliminate the need to align these sequences for species identification.

In an attempt to overcome these difficulties, we propose here a new species identification strategy taking advantage of both bioinformatics and machine learning as an extension of our prior back-propagation neural network application [15]. It is especially aimed at identifying species with non-coding barcodes, a topic little explored in the current barcoding literature. We test our methods with four empirical datasets, two representing typical protein-coding COI barcodes and two using the non-coding barcode ITS, and compare the results to those from two traditional barcoding strategies, Neighbor-joining (NJ) [44] and Maximum likelihood (ML) [62]. We used more than 21,220 random queries against the corresponding reference libraries. We demonstrate that the new procedures outperform the two traditional barcoding methods and BP-based methods [15]. This is largely because sequence alignments are no longer required - a big advantage for non-coding sequences - and to the saving of computational time compared to previous BP-based methods [15].

## Results

### Neotropical bat and Marine fish COI datasets

**Figure 1:**
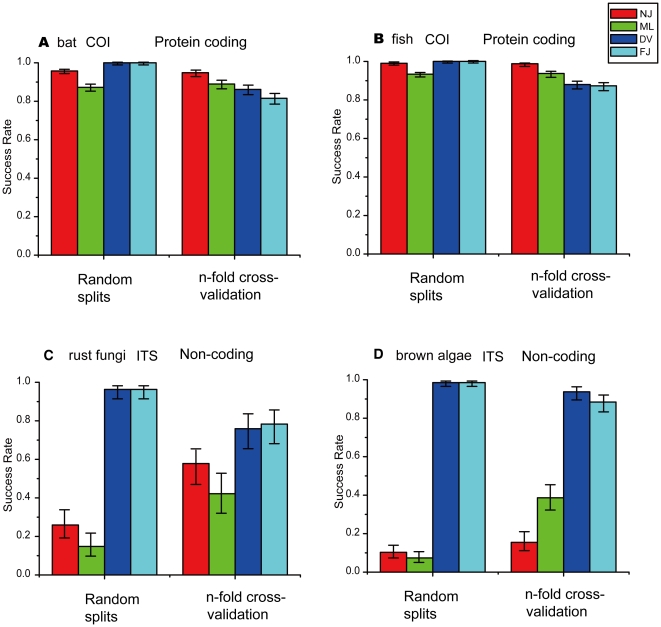
Success rate of species identification and 95% confidence intervals with the new methods (DV-RBF or FJ-RBF) proposed in this study based on COI barcodes and ITS barcodes for four empirical datasets.

In total, 8,120 random queries from 766 bat COI sequences were examined with two traditional methods (NJ and ML) and the two newly proposed methods (DV-RBF and FJ-RBF) against corresponding reference libraries. 5,180 of these queries were carried out using 5 repeated random splits, representing complete/balanced species coverage in the reference library (meaning that all species from the original database remain in the reference library, see Materials and Methods). The remaining 2,940 queries were conducted using five-fold cross-validation, representing incomplete/unbalanced species coverage in the reference library (meaning that some species from the original database might be absent from the reference library). For the two new methods, 4,122 queries were performed against the corresponding reference databases (Table 1, Table S1). In the case of balanced species coverage, both DV-RBF and FJ-RBF methods achieved 100% success rates (95% CI: 99.70–100%) with 1,295 random queries each (Figure 1a), while the NJ and ML methods obtained success rates of 95.75% (95% CI: 94.51–96.72%), and 87.25% (95% CI: 85.33–88.96%) respectively. For unbalanced species coverage, with 766 random queries for each of DV-RBF and FJ-RBF, the NJ method outperformed all other methods (94.86% with a 95% CI: 92.93–96.28%) compared with ML 88.97% (95% CI: 86.53–91.01%); DV-RBF 86.18% (95% CI: 83.53–88.46%) and FJ-RBF 81.54% (95% CI: 78.61–84.14%) (Figure 1a). The slightly better performance of ML than either DV-RBF and FJ-RBF was without statistical significance (Figure 1 1a).

**Table 1:** Species assignments for Neotropical bats [81] based on COI sequences for all 4122 random queries using DV-RBF and FJ-RBF methods.

No.	Category of Random Tests^a^	Query^b^	DV-RBF^c^	Status	FJ-RBF^d^	Status
1	random	BCBNT34706-Rhynchonycteris naso	*Rhynchonycteris naso*	(✓^e^)	*Rhynchonycteris naso*	(✓^e^)
2	splits	BCBNT35706-Rhynchonycteris naso	*Rhynchonycteris naso*	(✓)	*Rhynchonycteris naso*	(✓)
3		BCBNT13006-Diclidurus isabellus	*Diclidurus isabellus*	(✓)	*Diclidurus isabellus*	(✓)
4		BCBNT37906-Diclidurus isabellus	*Diclidurus isabellus*	(✓)	*Diclidurus isabellus*	(✓)
5		BCBNT14306-Diclidurus isabellus	*Diclidurus isabellus*	(✓)	*Diclidurus isabellus*	(✓)
6		BCBNT92206-Chrotopterus auritus	*Chrotopterus auritus*	(✓)	*Chrotopterus auritus*	(✓)
7		BCBNT59706-Chrotopterus auritus	*Chrotopterus auritus*	(✓)	*Chrotopterus auritus*	(✓)
8		BCBNT04006-Cormura brevirostris	*Cormura brevirostris*	(✓)	*Cormura brevirostris*	(✓)
9		BCBNT05606-Cormura brevirostris	*Cormura brevirostris*	(✓)	*Cormura brevirostris*	(✓)
10		BCBNT39906-Pteronotus personatus	*Pteronotus personatus*	(✓)	*Pteronotus personatus*	(✓)
11		BCBNT09706-Pteronotus personatus	*Pteronotus personatus*	(✓)	*Pteronotus personatus*	(✓)
12		BCBNT36906-Noctilio albiventris	*Noctilio albiventris*	(✓)	*Noctilio albiventris*	(✓)
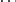		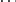	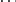	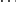	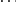	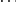
1295	( 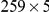 )	BCBNT55406-Lophostoma silvicolum	*Lophostoma silvicolum*	(✓)	*Lophostoma silvicolum*	(✓)
1	n-fold	BCBNT29806-Trachops cirrhosus	*Trachops cirrhosus*	(✓)	*Trachops cirrhosus*	(✓)
2	cross-	BCBNT63906-Platyrrhinus helleri	*Platyrrhinus helleri*	(✓)	*Platyrrhinus helleri*	(✓)
3	validation	BCBNC12906-Rhinophylla pumilio	*Rhinophylla pumilio*	(✓)	*Rhinophylla pumilio*	(✓)
4		BCBNT94306-Molossus molossus	*Molossus molossus*	(✓)	*Molossus molossus*	(✓)
5		BCBNC01906-Rhinophylla pumilio	*Rhinophylla pumilio*	(✓)	*Rhinophylla pumilio*	(✓)
6		BCBNT70306-Phyllostomus discolor	*Phyllostomus discolor*	(✓)	*Phyllostomus discolor*	(✓)
7		BCBNT99106-Platyrrhinus aurarius	*Platyrrhinus aurarius*	(✓)	*Carollia perspicillata*	(✗)
8		BCBNC16806-Platyrrhinus aurarius	*Platyrrhinus aurarius*	(✓)	*Platyrrhinus aurarius*	(✓)
9		BCBN31305-Rhinophylla pumilio	*Rhinophylla pumilio*	(✓)	*Rhinophylla pumilio*	(✓)
10		BCBNC06506-Lionycteris spurrelli	*Lionycteris spurrelli*	(✓)	*Lionycteris spurrelli*	(✓)
11		BCBN55205-Trachops cirrhosus	*Trachops cirrhosus*	(✓)	*Trachops cirrhosus*	(✓)
12		BCBNC16106-Platyrrhinus aurarius	*Platyrrhinus aurarius*	(✓)	*Platyrrhinus aurarius*	(✓)
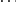		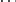	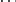	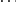	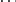	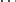
766	( 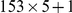 )	BCBNT94606-Glyphonycteris daviesi	*Carollia brevicauda*	(✗)	*Carollia perspicillata*	(✗)

a : Two categories of randomization were performed in this study. One is random splits which were conducted at species level (5 times) and the other is n-fold cross-validation which was performed on the whole dataset (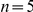 was used). 4122 random queries were generated based on the original 766 bat COI sequences, see text and Online Appendix I for details.

b : The names of query sequences consist of BOLD sequence accession numbers (a dash was removed before the last two numbers) and their true species names. Only part of the results were presented here, see Online Appendix I for all 4122 queries and corresponding assignments (singletons were excluded since they can only be assigned to the wrong speices).

c : DV denotes DV-Curve, RBF indicates RBF neural network, see text for details.

d : FJ denotes FJ-Curve.

e : Ticks and crosses indicate correct and wrong assignments respectively.

More than 10,000 (10,040) random replications of queries were performed for the fish dataset against the corresponding reference libraries. 6,340 random queries were carried out with 5 repeated random splits, representing complete/balanced species coverage. The remaining 3,700 random queries were assigned with five-fold cross-validation, representing incomplete/unbalanced species coverage in the reference library. For the two new methods, 5,134 queries were performed against the corresponding reference databases (Table 2, Table S2).

**Table 2:** Species assignments for Pacific Canadian marine fish [82] based on COI sequences for all 5134 random queries using DV-RBF and FJ-RBF methods.

No.	Category of Random Tests^a^	Query^b^	DV-RBF^c^	Status	FJ-RBF^d^	Status
1	random	TZFPA15007-Eptatretus stoutii	*Eptatretus stoutii*	(✓^e^)	*Eptatretus stoutii*	(✓^e^)
2	splits	TZFPB55006-Eptatretus stoutii	*Eptatretus stoutii*	(✓)	*Eptatretus stoutii*	(✓)
3		TZFPB57806-Eptatretus stoutii	*Eptatretus stoutii*	(✓)	*Eptatretus stoutii*	(✓)
4		TZFPB21505-Eptatretus deani	*Eptatretus deani*	(✓)	*Eptatretus deani*	(✓)
5		TZFPB32505-Eptatretus deani	*Eptatretus deani*	(✓)	*Eptatretus deani*	(✓)
6		TZFPB04605-Porichthys notatus	*Porichthys notatus*	(✓)	*Porichthys notatus*	(✓)
7		TZFPB46906-Porichthys notatus	*Porichthys notatus*	(✓)	*Porichthys notatus*	(✓)
8		TZFPB04305-Porichthys notatus	*Porichthys notatus*	(✓)	*Porichthys notatus*	(✓)
9		TZFPB53606-Squalus acanthias	*Squalus acanthias*	(✓)	*Squalus acanthias*	(✓)
10		TZFPB56706-Squalus acanthias	*Squalus acanthias*	(✓)	*Squalus acanthias*	(✓)
11		TZFPB55906-Squalus acanthias	*Squalus acanthias*	(✓)	*Squalus acanthias*	(✓)
12		TZFPB42505-Cyclothone atraria	*Cyclothone atraria*	(✓)	*Cyclothone atraria*	(✓)
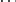		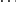	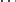	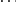	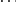	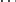
1585	( 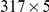 )	TZFPA19707-Malacocottus	*Malacocottus zonurus*	(✓)	*Malacocottus zonurus*	(✓)
1	n-fold	TZFPB55306-Lycodes diapterus	*Lycodes diapterus*	(✓)	*Lycodes diapterus*	(✓)
2	cross-	TZFPB69106-Sebastes pinniger	*Sebastes pinniger*	(✓)	*Sebastes pinniger*	(✓)
3	validation	TZFPA14506-Talismania bifurcata	*Talismania bifurcata*	(✓)	*Talismania bifurcata*	(✓)
4		TZFPB71206-Ronquilus jordani	*Ronquilus jordani*	(✓)	*Ronquilus jordani*	(✓)
5		TZFPB56606-Sebastes aleutianus	*Sebastes aleutianus*	(✓)	*Sebastes aleutianus*	(✓)
6		TZFPA19407-Nectoliparis pelagicus	*Oncorhynchus tshawytscha*	(✗)	*Bathyagonus infraspinatus*	(✗)
7		TZFPB82006-Sebastes reedi	*Sebastes reedi*	(✓)	*Sebastes reedi*	(✓)
8		TZFPB46706-Alosa sapidissima	*Alosa sapidissima*	(✓)	*Alosa sapidissima*	(✓)
9		TZFPB87508-Oligocottus maculosus	*Oligocottus maculosus*	(✓)	*Oligocottus maculosus*	(✓)
10		TZFPB32805-Alepocephalus tenebrosus	*Alepocephalus tenebrosus*	(✓)	*Alepocephalus tenebrosus*	(✓)
11		TZFPB58306-Theragra chalcogramma	*Theragra chalcogramma*	(✓)	*Theragra chalcogramma*	(✓)
12		TZFPB86908-Cyclothone atraria	*Sebastolobus alascanus*	(✗)	*Bathyagonus infraspinatus*	(✗)
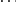		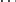	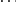	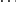	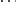	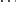
982	( 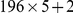 )	TZFPB16505-Sebastes flavidus	*Sebastes flavidus*	(✓)	*Sebastes flavidus*	(✓)

a : Two categories of randomization were performed in this study. One is random splits which were conducted at species level (5 times) and the other is n-fold cross-validation which was performed on the whole dataset (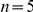 was used). 5134 random queries were generated based on the original 982 fish COI sequences, see text and Online Appendix II for details.

b : The names of query sequences consist of BOLD sequence accession numbers (a dash was removed before the last two numbers) and their true species names. Only part of the results were presented here, see Online Appendix II for all 5134 queries and corresponding assignments (singletons were excluded since they can only be assigned to the wrong speices).

c : DV denotes DV-Curve, RBF indicates RBF neural network, see text for details.

d : FJ denotes FJ-Curve.

e : Ticks and crosses indicate correct and wrong assignments respectively.

In the situation of complete species coverage, the two new methods (DV-RBF and FJ-RBF) had 100% success rates (95% CI: 99.75–100%), significantly outperforming the two traditional methods that gave success rates of 99.05% (95% CI: 98.44–99.42%) and 93.37 (95% CI: 92.04–94.49%) for NJ and ML respectively (Figure 1b). However, traditional NJ and ML approaches significantly outperformed both DV-RBF and FJ-RBF under the circumstance of unbalanced species coverage (Figure 1b) (NJ, 98.81% with 95% CI: 97.88–99.33%; ML, 93.72% with 95% CI:91.97–95.11% ; DV-RBF, 88.00% with 95% CI: 85.74–89.93%; FJ-RBF, 87.35% with 95% CI: 85.05–89.04%). Our results from the bat and fish protein-coding COI datasets showed that the structure of reference libraries (balanced versus unbalanced species coverage) could affect species identification success rates. The two newly proposed methods perform very well in the former situation, but less well in the latter.

### Rust fungi ITS dataset

Since ITS barcodes were only recently developed as alternative barcode markers, there are relatively limited data available. We obtained 85 clean sequences from 14 species of rust fungi and performed 872 random queries with the four barcoding methods. 540 queries were conducted under the situation of balanced species coverage (5 repeated random splits) and 332 queries for the case of unbalanced species coverage (five-fold cross validation, Table 3). The two new methods (DV-RBF and FJ-RBF) significantly outperformed the two traditional methods (NJ and ML) whether or not species coverage in the reference library is balanced (Figure 1c). For instance, both DV-RBF and FJ-RBF methods achieved a 96.29% success rate (95% CI: 91.62–98.40%) for unbalanced coverage while NJ and ML only obtained success rates of 25.92% (95% CI: 19.27–33.91%) and 14.81% (95% CI: 9.79–21.77%) respectively (Figure 1c). In the situation of balanced species coverage, traditional NJ and ML methods obtained higher but still less than 60.00% success rates (NJ, 57.00% with 95% CI: 47.09–67.87% ; ML, 42.16% with 95% CI: 32.12–52.90%; Figure 1c), again much less than the success rates for the two new methods (DV-RBF, 75.90% with 95% CI: 65.19–83.82%; FJ-RBF, 78.31% with 95% CI: 68.30–85.81; Figure 1c; Table S3). Thus in the case of non-coding barcodes, the two newly proposed methods (DV-RBF and FJ-RBF) considerably outperformed the two traditional methods (NJ and ML) regardless of the structure of reference libraries (balanced versus unbalanced species coverage).

**Table 3:** Species assignments for rust fungi (BOLD project CHITS) based on ITS sequences for 484 random queries using DV-RBF and FJ-RBF methods.

No.	Category of Random Tests^a^	Query^b^	DV-RBF^c^	Status	FJ-RBF^d^	Status
1	random	CHITS08008-Chrysomyxa wereii	*Chrysomyxa wereii*	(✓^e^)	*Chrysomyxa wereii*	(✓^e^)
2	splits	CHITS07708-Chrysomyxa wereii	*Chrysomyxa wereii*	(✓)	*Chrysomyxa wereii*	(✓)
3		CHITS11109-Chrysomyxa pirolata	*Chrysomyxa pirolata*	(✓)	*Chrysomyxa pirolata*	(✓)
4		CHITS01308-Chrysomyxa pirolata	*Chrysomyxa pirolata*	(✓)	*Chrysomyxa pirolata*	(✓)
5		CHITS11009-Chrysomyxa pirolata	*Chrysomyxa pirolata*	(✓)	*Chrysomyxa pirolata*	(✓)
6		CHITS09509-Chrysomyxa arctostaphyli	*Chrysomyxa arctostaphyli*	(✓)	*Chrysomyxa arctostaphyli*	(✓)
7		CHITS04108-Chrysomyxa arctostaphyli	*Chrysomyxa arctostaphyli*	(✓)	*Chrysomyxa arctostaphyli*	(✓)
8		CHITS03208-Chrysomyxa empetri	*Chrysomyxa empetri*	(✓)	*Chrysomyxa empetri*	(✓)
9		CHITS03308-Chrysomyxa empetri	*Chrysomyxa empetri*	(✓)	*Chrysomyxa empetri*	(✓)
10		CHITS03108-Chrysomyxa chiogenis	*Chrysomyxa chiogenis*	(✓)	*Chrysomyxa chiogenis*	(✓)
11		CHITS02408-Chrysomyxa chiogenis	*Chrysomyxa chiogenis*	(✓)	*Chrysomyxa chiogenis*	(✓)
12		CHITS06208-Chrysomyxa ledicola	*Chrysomyxa ledicola*	(✓)	*Chrysomyxa ledicola*	(✓)
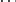		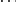	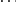	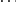	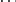	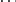
135	( 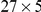 )	CHITS06508-Chrysomyxa nagodhii	*Chrysomyxa nagodhii*	(✓)	*Chrysomyxa nagodhii*	(✓)
1	n-fold	CHITS05608-Chrysomyxa ledi	*Chrysomyxa rhododendri*	(✗)	*Chrysomyxa rhododendri*	(✗)
2	cross-	CHITS01208-Chrysomyxa cassandrae	*Chrysomyxa cassandrae*	(✓)	*Chrysomyxa cassandrae*	(✓)
3	validation	CHITS04008-Chrysomyxa arctostaphyli	*Chrysomyxa arctostaphyli*	(✓)	*Chrysomyxa arctostaphyli*	(✓)
4		CHITS02308-Chrysomyxa chiogenis	*Chrysomyxa chiogenis*	(✓)	*Chrysomyxa chiogenis*	(✓)
5		CHITS02108-Chrysomyxa nagodhii	*Chrysomyxa cassandrae*	(✗)	*Chrysomyxa ledi*	(✗)
6		CHITS05308-Chrysomyxa arctostaphyli	*Chrysomyxa ledicola*	(✗)	*Chrysomyxa ledi*	(✗)
7		CHITS06208-Chrysomyxa ledicola	*Chrysomyxa ledicola*	(✓)	*Chrysomyxa ledicola*	(✓)
8		CHITS06008-Chrysomyxa ledicola	*Chrysomyxa ledicola*	(✓)	*Chrysomyxa ledicola*	(✓)
9		CHITS09509-Chrysomyxa arctostaphyli	*Chrysomyxa ledicola*	(✗)	*Chrysomyxa ledi*	(✗)
10		FUCUI00608-Fucus distichus	*Fucus distichus*	(✓)	*Fucus distichus*	(✓)
11		CHITS11009-Chrysomyxa pirolata	*Chrysomyxa pirolata*	(✓)	*Chrysomyxa pirolata*	(✓)
12		CHITS05708-Chrysomyxa ledi	*Chrysomyxa rhododendri*	(✗)	*Chrysomyxa rhododendri*	(✗)
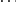		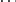	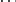	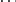	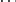	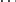
107	( 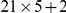 )	CHITS08909-Chrysomyxa ledicola	*Chrysomyxa ledicola*	(✓)	*Chrysomyxa ledicola*	(✓)

a : Two categories of randomization were performed in this study. One is random splits which were conducted at species level (5 times) and the other is n-fold cross-validation which was performed on the whole dataset (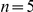 was used). 484 random queries were generated based on the original 107 rust fungi ITS sequences, see text and Online Appendix III for details.

b : The names of query sequences consist of BOLD sequence accession numbers (a dash was removed before the last two numbers) and their true species names. Only part of the results were presented here, see Online Appendix III for all 484 queries and corresponding assignments (singletons were excluded since they can only be assigned to the wrong speices).

c : DV denotes DV-Curve, RBF indicates RBF neural network, see text for details.

d : FJ denotes FJ-Curve.

e : Ticks and crosses indicate correct and wrong assignments respectively.

### Brown algae ITS dataset

207 ITS sequences of brown algae data from 16 species were obtained after data cleansing. We performed 2,188 random queries against corresponding reference libraries with the four barcoding methods, of which 1,360 were conducted using repeated random splits (5 times, each 340 queries for each method), and 828 using five-fold cross-validation (Table 4 and Table S4). As in the case of the rust fungi ITS dataset, both DV-RBF and FJ-RBF methods outperformed with statistical significance the two traditional methods (Figure 1d). A success rate of 98.52% (95% CI: 96.60–99.37%) was achieved for both DV-RBF and FJ-RBF methods in the case of balanced species coverage while NJ and ML methods obtained extremely low success rates of 10.29% (95% CI: 7.49–13.98%) and 7.35% (95% CI: 5.02–10.62%) respectively (Figure 1d). In the situation of unbalanced species coverage, DV-RBF and FJ-RBF obtained somewhat reduced success rates (DV-RBF, 93.71% with 95% CI: 89.55–96.29%; FJ-RBF, 88.40% with 95% CI: 83.32–92.08%), but they were nevertheless much larger than those of the two traditional methods (NJ, 15.45% with 95% CI: 11.16–21.00%; ML, 38.64% with 95% CI: 32.27–45.43%; Figure 1d).

**Table 4:** Species assignments for the brown algae (BOLD project PHAEP) based on ITS sequences for 1094 random queries using DV-RBF and FJ-RBF methods.

No.	Category of Random Tests^a^	Query^b^	DV-RBF^c^	Status	FJ-RBF^d^	Status
1	random	FUCUI04008-Fucus distichus	*Fucus distichus*	(✓^e^)	*Fucus distichus*	(✓^e^)
2	splits	FUCUI03708-Fucus distichus	*Fucus distichus*	(✓)	*Fucus distichus*	(✓)
3		FUCUI04408-Fucus distichus	*Fucus distichus*	(✓)	*Fucus distichus*	(✓)
4		FUCUI03408-Fucus distichus	*Fucus distichus*	(✓)	*Fucus distichus*	(✓)
5		FUCUI00308-Fucus distichus	*Fucus distichus*	(✓)	*Fucus distichus*	(✓)
6		FUCUI05508-Fucus distichus	*Fucus distichus*	(✓)	*Fucus distichus*	(✓)
7		FUCUI02608-Fucus distichus	*Fucus distichus*	(✓)	*Fucus distichus*	(✓)
8		FUCUI04508-Fucus distichus	*Fucus distichus*	(✓)	*Fucus distichus*	(✓)
9		FUCUI05708-Fucus distichus	*Fucus distichus*	(✓)	*Fucus distichus*	(✓)
10		FUCUI04608-Fucus distichus	*Fucus distichus*	(✓)	*Fucus distichus*	(✓)
11		FUCUI02708-Fucus distichus	*Fucus distichus*	(✓)	*Fucus distichus*	(✓)
12		FUCUI00108-Fucus distichus	*Fucus distichus*	(✓)	*Fucus distichus*	(✓)
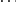		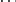	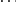	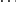	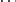	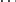
340	( 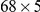 )	MACRO97608-Scytosiphon cylindricus	*Scytosiphon cylindricus*	(✓)	*Scytosiphon cylindricus*	(✓)
1	n-fold	MACRO69407-Saccharina latissima	*Saccharina latissima*	(✓)	*Saccharina latissima*	(✓)
2	cross-	MACRO12106-Saccharina latissima	*Saccharina latissima*	(✓)	*Saccharina latissima*	(✓)
3	validation	MACRO77607-Scytosiphon sp	*Scytosiphon sp*	(✓)	*Scytosiphon sp*	(✓)
4		MACRO11406-Scytosiphon cylindricus	*Scytosiphon cylindricus*	(✓)	*Scytosiphon cylindricus*	(✓)
5		MACRO12806-Scytosiphon cylindricus	*Scytosiphon cylindricus*	(✓)	*Scytosiphon cylindricus*	(✓)
6		MACRO49807-Saccharina latissima	*Saccharina latissima*	(✓)	*Saccharina latissima*	(✓)
7		MACRO17406-Petalonia sp	*Petalonia sp*	(✓)	*Petalonia sp*	(✓)
8		MACRO94108-Petalonia sp	*Petalonia sp*	(✓)	*Petalonia sp*	(✓)
9		FUCUI05308-Fucus spiralis	*Fucus spiralis*	(✓)	*Fucus spiralis*	(✓)
10		FUCUI00608-Fucus distichus	*Fucus distichus*	(✓)	*Fucus distichus*	(✓)
11		MACRO104108-Saccharina latissima	*Saccharina latissima*	(✓)	*Saccharina latissima*	(✓)
12		MACRO73607-Scytosiphon cylindricus	*Scytosiphon cylindricus*	(✓)	*Scytosiphon cylindricus*	(✓)
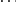		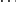	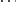	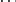	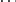	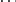
207	( 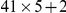 )	FUCUI00708-Fucus distichus	*Fucus distichus*	(✓)	*Fucus distichus*	(✓)

a : Two categories of randomization were performed in this study. One is random splits which were conducted at species level (5 times) and the other is n-fold cross-validation which was performed on the whole dataset (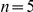 was used). 1094 random queries were generated based on the original 207 brown algae ITS sequences, see text and Online Appendix IV for details.

b : The names of query sequences consist of BOLD sequence accession numbers (a dash was removed before the last two numbers) and their true species names. Only part of the results were presented here, see Online Appendix IV for all 1094 queries and corresponding assignments (singletons were excluded since they can only be assigned to the wrong speices).

c : DV denotes DV-Curve, RBF indicates RBF neural network, see text for details.

d : FJ denotes FJ-Curve.

e : Ticks and crosses indicate correct and wrong assignments respectively.

### Processing time

The data analyses in this study were performed on a 3.00 GHz desktop computer (Intel(R) Core (TM)2, DuoCPU, E8400 @ 3.00 GHz×2). DV-RBF and FJ-RBF each spent 2.88-7.56 seconds per assignment, while the ML method spent 6.75–9.08 seconds per assignment exclusive of alignment time, depending on dataset size (from 68 to 785 reference sequences in this study). NJ spent less than one second per assignment, but the necessary sequence alignments can take several hours for a few hundred sequences.

## Discussion

The new methods proposed in this study for barcode-based species assignations, which combine bioinformatics and machine learning, provide several advantages over existing methods, including the earlier BP-based method [15].

The first advantage is that no sequence alignment is required. Alignment algorithms and interpretations have been highly debated topics in the field of evolutionary studies over the past several decades [63]–[66]. This reflects the difficulties faced in aligning homologs, especially from variable-length non-coding gene regions [64]. Most of the commonly used DNA barcoding approaches to species identification, including classical phylogenetic approaches such as neighbour joining [1], [2], [44], and decision theory [11] and Bayesian methods [36], [38], [39], rely heavily on an initial robust alignment. Our new methods circumvent this complex issue by taking advantage of graphical representations of DNA sequences via a DV-Curve [67] or the newly-developed (herein) FJ-Curve approach. We demonstrated their successful applications to four empirical datasets, two of which are based on the commonly used coding COI barcodes, and two on the more-recently proposed non-coding ITS barcodes. The new methods strongly outperformed the existing Neighbor-joining (NJ) and Maximum likelihood (ML) methods for non-coding barcodes, while the latter two performed slightly better than the new methods for coding barcodes in circumstances of potentially unbalanced species coverage in the reference library. The very large discrepancy in success between the traditional and the new methods proposed here in identifying species by ITS sequences is largely attributable to the former, especially the model-based methods, relying heavily on molecular evolutionary models which generally ignore the evolution of indels/gaps. The phylogenetic signals contained in the indels/gaps will be lost during the analysis. In the case of balanced species coverage in the reference database, the new methods outperformed the traditional NJ and ML methods for both coding and non-coding barcodes. This indicates that a complete reference library with balanced species coverage will improve species identification success rates: a well-curated reference database is an essential prerequisite for accurate species identification.

The second advantage, like the BP-based method [15], is that the new methods are based on fewer assumptions when making inferences. Most other current methods rely on a number of more or less restrictive assumptions that may not apply to real data [15], [36]. For example, the decision theory method [11] assumes an ideal panmictic population for all species or groups without recombination, migration, and so on, so that the evolutionary process within each group is governed by only one parameter: the number of mutational steps between two individuals within that group [15].

Whether it is worthwhile to adopt a biological, populational and/or phylogenetic rationale for DNA barcode sequence assignation, or whether pure statistical approaches are more efficient, remains largely unaddressed [68]. Species identifications via DNA barcoding can be complex both in theory and in practice [19]. Some authors [58] have argued that no one method can perform equally well in all circumstances of DNA barcoding. Machine learning based approaches [15], [45] which are neither classical population nor phylogeny based approaches, present fresh insights. The newly developed method here may be thought of as an extension of BP-based species identification [15], in the sense that both are based on machine learning, but it uses entirely different algorithms that apply the power of both bioinformatics and RBF neural networks (NN). The reason for choosing RBF NN is that it has been shown to work well when there are complex or highly non-linear relationships and relatively small training sets, which is the case for the sophisticated process of species assignments from DNA sequences. When the input data to an algorithm is too large to be processed, then the input data will be transformed into a reduced representation set of features (termed the features vector). Transforming the input data into the set of features is termed feature extraction. In DNA sequences, each site is treated as a feature. A simple n-gram approach is also commonly used for creating feature vectors, but this proved to be five times slower than NN methods in text categorization classification [69]. In Zhang et al. [15], DNA sequences were digitized simply using the codes A-0.1, T-0.2, G-0.3, C-0.4, and this proved to be successful. However, the converted input matrices are so huge that the training of NN becomes quite slow especially for large datasets. Both the DV-Curve and the FJ-Curve substantially reduce the data matrix dimensions from, for example, the 648 of standard COI barcodes to 24 (DV-Curve) or less (FJ-Curve). This property greatly improves computational speed when processing large datasets compared to BP-based species identification [15].

We also note that while this is a powerful approach, and one that is especially well suited for non-coding sequences such as ITS, it is not without problems. Like most currently used barcoding methods, it will assign a query to “the most like” species when the true species is not represented in the reference library. The issue of an incomplete reference database has been well explored in Ross *et al*. [40] and Ekrem *et al*. [70]. A new fuzzy set based species identification protocol has shed some light on this issue [71]. Unlike the BP-based method [15], the second limitation of the new method is that neither DV-RBF nor FJ-RBF approaches have the potential to incorporate non-DNA data into the system. Where several different sources of data are available, such as morphological characters or behavioural data, we would instead suggest using the BP-based approach proposed earlier [15].

## Materials and Methods

### Graphical Representation of DNA Sequences via Bioinformatic Approaches

#### The DV-Curve

**Figure 2:**
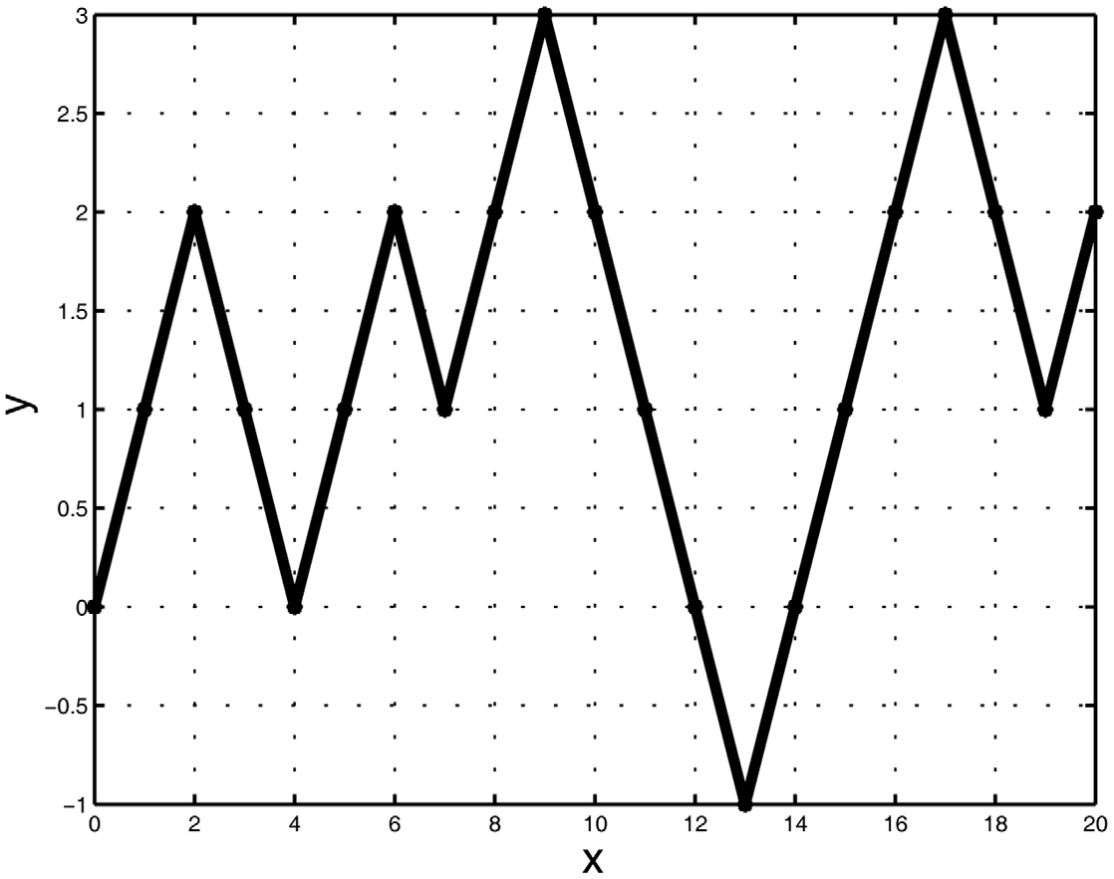
The DV-Curve of the 10 bp sequence ‘AGACTGCATC’.

The DV-Curve (Dual-Vector Curve) was proposed by Zhang [67] as a 2D graphical representation for the visualization and analysis of DNA sequences (Figure 2). It proved to be a good visualization for representing DNA sequences without degeneracy and loss of information. Let us consider a DNA sequence 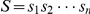 consisting of 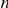 nucleotide sites. Let 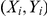 be the point of the DV-Curve, where 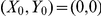 is the start point. The DV-Curve is uniquely determined by the following formula [67]:
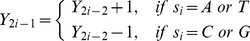
(1)

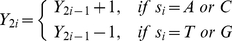
(2)

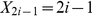
(3)

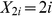
(4)where 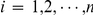.

#### The FJ-Curve

In this section, motivated by Jeffrey's ingenious work of chaos game representation (CGR) of DNA sequences [72], we propose a 3D representation of DNA sequences. Let 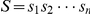 be a DNA sequence, 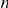 is the length of 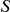. First we assign the four nucleotides to the four corners of a regular tetrahedron, i.e. A, G, C, T are assigned coordinates (−1, 1, −1), (1, 1, 1), (1, −1, −1) and (−1, −1, 1) respectively. Then we construct a curve for the given DNA sequence 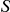. The point 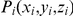 corresponding to 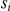 is calculated by the following formula:
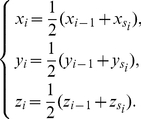
(5)

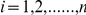
, 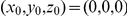 and 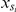, 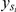 and 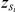 are calculated by the following formula:
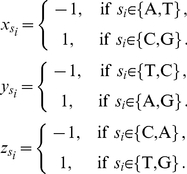
(6)In this way, 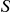 is converted into a series of points 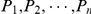. Let the origin 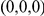 be the point 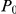. As the index 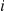 runs from 0 to 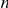, we connect the points 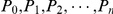 in turn and get a zigzag 3-D curve within a regular tetrahedron. This is the FJ-Curve of DNA sequence 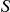 (named after one of the Authors (Dr. Feng Jie) of this study).

**Figure 3:**
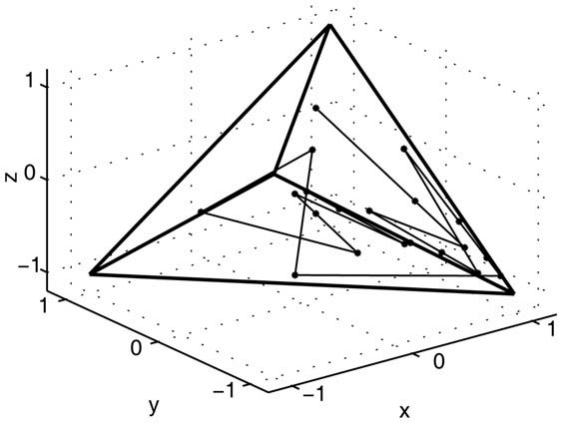
The FJ-Curve of the 20 bp sequence ‘GCCTCCGCCCAGACTTCTTC’.

From the FJ-Curve, some information on the base distribution and composition of the DNA sequence can be intuitively gathered. As an example, the FJ-curve for the twenty base length sequence GCCTCCGCCCAGACTTCTTC is shown in Figure 3. It is evident that most points are located near the vertex C (1, −1, −1), a consequence of the high proportion of C content in the sequence. On the other hand, because the A content is the lowest, the points near the vertex A are sparse.

#### Numerical Characterizations of the DV-Curve and the FJ-Curve

To numerically characterize a DNA sequence via the DV-Curve, a 24-component vector 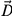 as described by Zhang [67] was used:
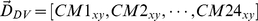
(7)


The 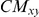 value [73] is calculated as follows:
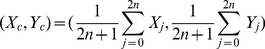
(8)

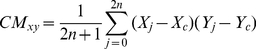
(9)


To get equation (7), we need to assign 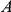,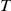,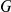,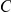 to basic Dual-Vectors in 4! different ways to have 4! = 24 different DV-Curves for a given DNA sequence. The vector 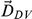 is further used as the input for a neural network.

We derive a set of numerical characterizations from the FJ-Curve of the DNA sequence as sequence descriptors: 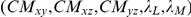. The first three descriptors [74] are calculated as follows:
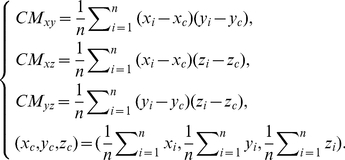
(10)The fourth descriptor is selected from the L/L matrix [74], in which the elements 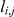 are defined as the quotient of the Euclidean distance between a pair of vertices (dots) of the FJ-Curve and the sum of distances between the same pair of vertices measured along the characteristic curve. In other words
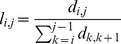
(11)where 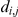 is the Euclidean distance between a pair of vertices. 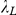 denotes the leading eigenvalue of the L/L matrix. The last descriptor is selected from the M/M matrix [75], in which the elements 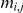 are given as the quotient of the Euclidean distance between two vertices of the FJ-Curve and the graph theoretical distance between the two vertices. In other words
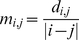
(12)where 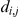 is the Euclidean distance between a pair of vertices. 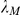 denotes the leading eigenvalue of the M/M matrix. To maximally extract information from DNA sequences, we here used both L/L and M/M matrices so there may be some overlap in information (i.e., redundant information) in the matrix representations. We therefore applied Principal Component Analysis (PCA) [76] to the matrix representations in order to reduce the correlation between L/L and M/M matrices. Principal Components whose contributions to total variation are less than 0.01 were ignored in the subsequent analysis.

### Radial Basis Function Neural Network

**Figure 4:**
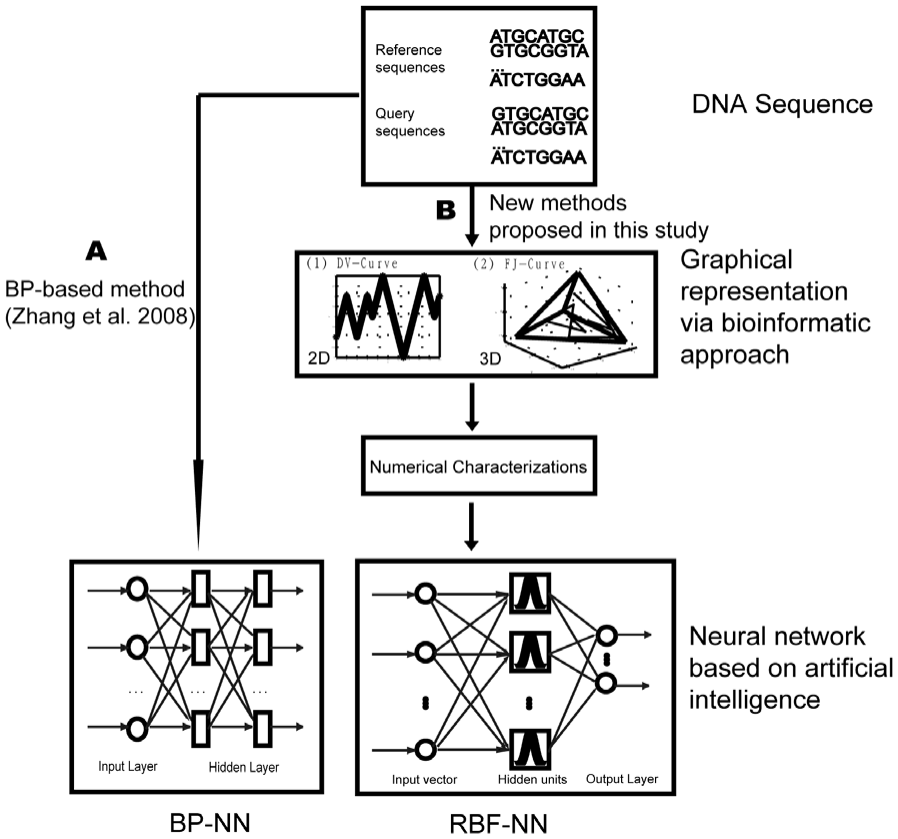
The work flow of the RBF network approach proposed in this study and a comparison with the BP network.

**Figure 5:**
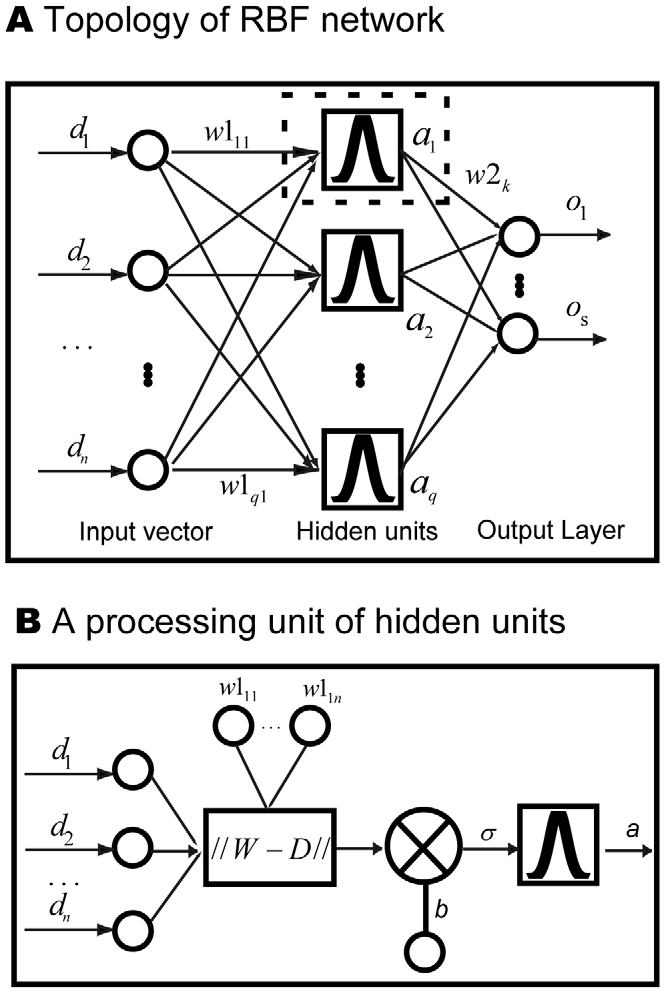
Topology of the RBF network and a processing unit of hidden units.

The BP-Neural Network was initially proposed by Zhang and colleagues [15], [45] to identify species in DNA barcoding and proved to be successful in both computer simulations and empirical studies. However, two inherent drawbacks of the BP-Neural Network preclude its wide application to DNA barcoding campaigns: its slow training for large reference datasets and potential local minimization during network training. In this study, we propose to use the Radial Basis Function (RBF) neural network which creates a network with zero error on training vectors. RBFs are embedded in a two-layer feed-forward neural network that is characterized by a set of inputs and outputs (Figure 4 and 5). Between the inputs and outputs there is a layer of processing units called hidden units, each of which implements a radial basis function [77].

The Gaussian activation function for RBF network is given by
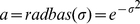
(13)


The input of hidden units is given by
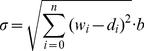
(14)where 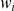 is the input vector and 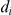 is the weight of hidden units.

We, therefore, have
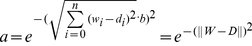
(15)


The output layer implements a weighted sum of hidden-unit outputs:
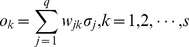
(16)


### Species Identification via DNA sequences in DNA Barcoding

#### Training a network using reference sequences

Instead of simply encoding the raw DNA sequences as inputs of a neural network [15], we here employed both bioinformatic approaches (the DV-Curve and the FJ-Curve) and machine learning for species identification. The numerical characterizations of the DV-Curves and the FJ-Curves computed earlier were fed into the RBF neural networks as inputs. The former is termed the DV-Curve based RBF (DV-RBF) and the latter the FJ-Curve based RBF (FJ-RBF). The network takes a matrix of input vectors 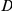 and target vectors 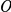. The training will return a network with weights and biases 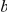 such that the outputs 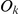 are exactly 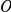 when the inputs are 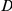. Generally, during network training, the weights of hidden units are set to 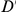 and each bias in the hidden units is set to a value which is determined by the width of an area in the input space to which each neuron responds. The second-layer (output layer) weights and biases are computed by simulating the first-layer outputs 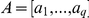, and then solving a linear expression
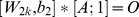
(17)


Since the inputs to the second-layer 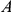 and the target (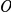) and the layer are linear, we can use the following formula to calculate the weights and biases of the output layer to minimize the sum-squared error:
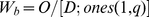
(18)where 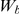 contains both weights and biases, with the biases in the last column, and 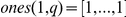.

#### Identifying query sequences using a trained network

The query sequences were firstly transferred into a numeral matrix using the method described above (the DV-Curve or the FJ-Curve), which served as the input vector (Figs. 4 and 5). Then, the input vector was fed into the trained network, and one output row vector, corresponding to a different species following the formula of Zhang *et al.* [15], was obtained for each input vector. The output vector of the network for one sequence selected from, for example, species 1, could be like ‘
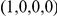
’ in the case of four species in the reference library through activation of the competitive function.

### Success Rate of Species Identification and Confidence Intervals

The success rate of species identification is defined as the following formula [15]:
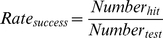
(19)


Binary data indicating the presence (successful identification) or absence (failed identification) of a specific attribute are often modeled as random samples from a Bernoulli distribution with parameter 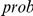, where 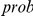 is the proportion in the population with that attribute. A 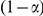-level confidence interval (CI) for 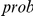 is calculated by the following formula [78]:
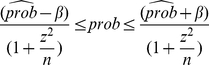
(20)where 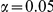,
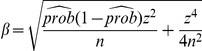
, 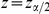.

### Comparison to the Existing Methods with Repeated Random Splits and n-fold Cross-validation

We wished to determine how our new methods for species identification compare with traditional DNA barcoding approaches, including Neighbor-joining (NJ) [44] and Maximum likelihood [62]. We did this utilising both repeated random splits and n-fold cross-validation. There are some differences between these two randomization strategies. For the former, sequences from an original empirical dataset were divided into two sub-datasets: a reference set and a query set. The reference set comprised all sequences from species with samples of 3 or less, together with two thirds (or as close as possible) of sequences from species with samples of 4 or greater. Remaining sequences formed the query set. This process was repeated 5 times. The resultant reference set has a ‘complete/balanced’ species coverage since random splits were performed at the level of species, and all species in the original database will be kept in the reference library. In n-fold cross-validation, data were split into 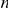 partitions and a subset from the 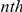 partition used to validate the success rate estimated from the remaining data. We here used a five-fold cross-validation to examine all methods under study. The subsequent reference library will have an ‘incomplete/unbalanced’ species coverage since the random partition was conducted on the whole original dataset, and therefore not all species are guaranteed to be included in the reference library. For the two traditional methods, each query from the query set was selected to form a new dataset with all reference sequences, and a genetic distance matrix was generated with the K2P model [79]. An NJ tree was then constructed with PAUP*beta [80] and an ML tree built with PHYML [62]. A successful identification was counted when a query fell into a monophyletic species clade. Species identification success rates were estimated over all random queries and 95% CI estimated with equation (20). For simplicity, the success rate from all 5-fold cross-validations was combined for the confidence interval estimate, although the pooling of results from 5-fold cross-validations could underestimate the CI. However, this underestimation was treated as trivial in this study since sampling sizes were generally large (much larger than 30). For all methods, singletons in the query set not represented in the reference set were not counted when calculating success rates, since these singletons would necessarily be assigned to the wrong species.

### COI Datasets

#### Neotropical Bat Dataset and Marine Fish Dataset

The COI dataset of 87 Neotropical species from 47 genera of bat in Guyuna contained 819 COI sequences with lengths greater than 600 bp [81]. These were downloaded from the Barcode of Life Database (BOLD, www.barcodinglife.org) on May 10, 2010. We cleaned the dataset by removing sequences with ambiguous sites, such as “Ns”, and those whose length were less than 648 bp (the standard length in COI DNA barcoding) [1]–[4]. This gave 766 COI sequences from 84 species. The second COI dataset was North Pacific fish. Steinke *et al* barcoded 201 North Pacific fish species, yielding 1225 barcode sequences [82]. We downloaded these from BOLD project TZFPC, Fishes of Pacific Canada Part I, on May 10, 2010. Read lengths were about 655 bp long. To reduce the effect of ambiguous sites on the analysis, we again filtered the dataset by removing uncertain nucleotide sites, such as “Ns”. The 982 resultant 652 bp alignments were used in the subsequent analysis. Meanwhile, five-fold cross-validation was performed as well (Table 1 and 2).

### ITS Datasets

#### Rust fungi dataset and Brown algae dataset

The rust fungi dataset comprised 108 ITS sequences from 16 species in BOLD (project CHITS, Chrysomyxa ITS Barcoding; downloaded on June 4, 2010). The length of these sequences varied from 625 bp to 792 bp, excepting one sequence of 333 bp. The dataset was cleaned as above by removing sequences with ambiguous sites (e.g. ‘Ns’). 85 sequences representing 14 species remained for the subsequent analysis. An initial alignment of the sequences was made using the program MUSCLE [83] with the default setting to check the homology of the sequences as a whole. All the indels (gaps) introduced during the alignment were eliminated later, the sequences for the subsequent analysis thus remained unaligned. 216 ITS sequences belonging to 20 species from seven genera of brown algae were retrieved from BOLD (project PHAEP; Phaeophyceae published, downloaded on June 20, 2010). These sequences cover a broad diversity of brown algae (six families from four orders). Sequences containing ambiguous sites were removed, and the resultant 207 sequences were highly variable in length (387 bp to 1215 bp).

## Supporting Information

Table S1 Species assignments for Neotropical bats [81] based on COI sequences for all 4122 random queries using DV-RBF and FJ-RBF methods in details.
(XLS)

Table S2 Species assignments for Pacific Canadian marine fish [82] (BOLD project TZFPC) based on COI sequences for all 5134 random queries using DV-RBF and FJ-RBF methods in details.
(XLS)

Table S3 Species assignments for rust fungi (BOLD project CHITS) based on ITS sequences for 484 random queries using DV-RBF and FJ-RBF methods in details.
(XLS)

Table S4 Species assignments for the brown algae (BOLD project PHAEP) based on ITS sequences for 1094 random queries using DV-RBF and FJ-RBF methods in details.
(XLS)
